# Crystal structure of *N*,*N*′-bis­(pyridin-3-ylmeth­yl)cyclo­hexane-1,4-di­ammonium dichloride

**DOI:** 10.1107/S2056989016017205

**Published:** 2016-11-04

**Authors:** Suk-Hee Moon, Hansu Im, Tae Ho Kim, Ki-Min Park

**Affiliations:** a Department of Food and Nutrition, Kyungnam College of Information and Technology, Busan 47011, Republic of Korea; b Research institute of Natural Science and Department of Chemistry, Gyeongsang National University, Jinju 52828, Republic of Korea

**Keywords:** crystal structure, diprotonated structure, dipyridyl salt, hydrogen bonding, condensation reaction

## Abstract

The title salt, C_18_H_26_N_4_^2+^·2Cl^−^, was obtained by the protonation of *N*,*N*-bis­(pyridin-4-ylmeth­yl)cyclo­hexane-1,4-di­amine with hydro­chloric acid in ethanol. The asymmetric unit consists of one half of an *N*,*N*-bis­(pyridin-3-ylmeth­yl)cyclo­hexane-1,4-di­ammonium dication, with a crystallographic inversion centre located at the centre of the cyclo­hexyl ring, and a chloride anion. The central cyclo­hexyl ring in the dication adopts a chair conformation. The two *trans*-(4-pyridine)–CH_2_–NH_2_– moieties at the 1- and 4-positions of the central cyclo­hexyl ring occupy equatorial sites. The terminal pyridine ring is tilted by 53.72 (6)° with respect to the mean plane of the central cyclo­hexyl ring (r.m.s. deviation = 0.2413 Å). In the crystal, N^+^—H⋯Cl^−^ hydrogen bonds between the dications and the chloride anions, and π–π stacking inter­actions between the pyridine rings of the dications afford a two-dimensional sheet extending parallel to the *ab* plane. These sheets are further connected through weak C—H⋯Cl^−^ hydrogen bonds, resulting in the formation of a three-dimensional supra­molecular network.

## Abstract 2

The title salt, C_18_H_26_N_4_^2+^·2Cl^−^, is located about a crystallographic inversion centre at the centre of the cyclo­hexyl ring, which adopts a chair conformation. In the crystal, N^+^(C)—H⋯Cl^−^ hydrogen bonds and π–π stacking inter­actions lead to the formation of a three-dimensional supra­molecular network.

## Chemical context   

Several dipyridyl-type ligands with or without a central section between the terminal pyridine rings have contributed greatly to the development of metal–organic coordination polymers with intriguing topologies or potential applications (Silva *et al.*, 2015 ▸; Furukawa *et al.*, 2014 ▸; Robin & Fromm, 2006 ▸; Robson, 2008 ▸; Leong & Vittal, 2011 ▸). Our group has also tried to prepare extended dipyridyl-type ligands with a bulky central moiety for the construction of versatile coordination polymers. Recently, we prepared the dipyridyl-type ligand containing 4-pyridine terminal groups and a cyclo­hexyl ring as a bulky central moiety, namely *N*,*N*-bis­(pyridin-4-ylmeth­yl)cyclo­hexane-1,4-di­amine, and reported the crystal structure of its chloride salt (Moon *et al.*, 2016 ▸). As an extension of our research, we have prepared a dipyridyl-type ligand with central cyclo­hexyl ring and 3-pyridine terminal groups, namely *N*,*N*-bis­(pyridin-3-ylmeth­yl)cyclo­hexane-1,4-di­amine, synthesized by a condensation reaction between 1,4-cyclo­hexa­ne­diamine and 3-pyridine­carboxaldehyde according to the literature procedure (Huh & Lee, 2007 ▸). Herein we report on crystal structure of the title salt obtained by the protonation of both amine groups in this mol­ecule.

## Structural commentary   

**Figure 1:**
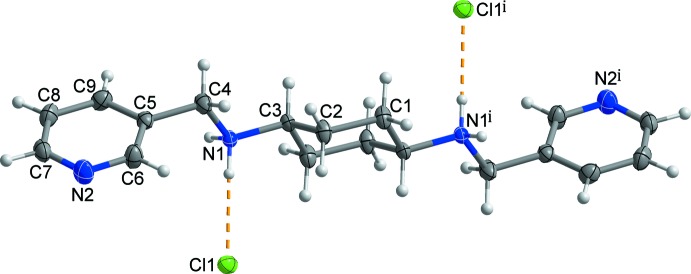
A view of the mol­ecular structure of the title salt, showing the atom-numbering scheme. Displacement ellipsoids are drawn at the 50% probability level. H atoms are shown as small spheres of arbitrary radius and yellow dashed lines represent the inter­molecular N^+^—H⋯Cl^−^ hydrogen bonds. [Symmetry code: (i) −*x* + 1, −*y* + 1, −*z* + 1.]

Fig. 1 ▸ shows the mol­ecular structure of the title salt, which lies about an inversion centre located at the centre of the cyclo­hexyl ring. Therefore, the asymmetric unit comprises one half of the *N*,*N*-bis­(pyridin-3-ylmeth­yl)cyclo­hexane-1,4-di­ammo­nium dication and a chloride anion. In the dication, the central cyclo­hexyl ring displays a chair conformation and the two *trans*-(4-pyridine)–CH_2_–NH_2_– moieties occupy equatorial sites at the 1- and 4-positions of the central cyclo­hexyl ring. The terminal pyridine ring is tilted by 53.72 (6)° with respect to the mean plane of the cyclo­hexyl ring (r.m.s. deviation = 0.2413 Å). This tilting angle is larger than that [27.98 (5)°] of the similar dication with 4-pyridine rings as the terminal groups (Moon *et al.*, 2016 ▸).
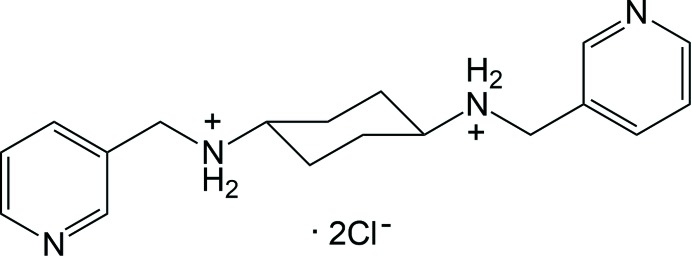


## Supra­molecular features   

**Figure 2:**
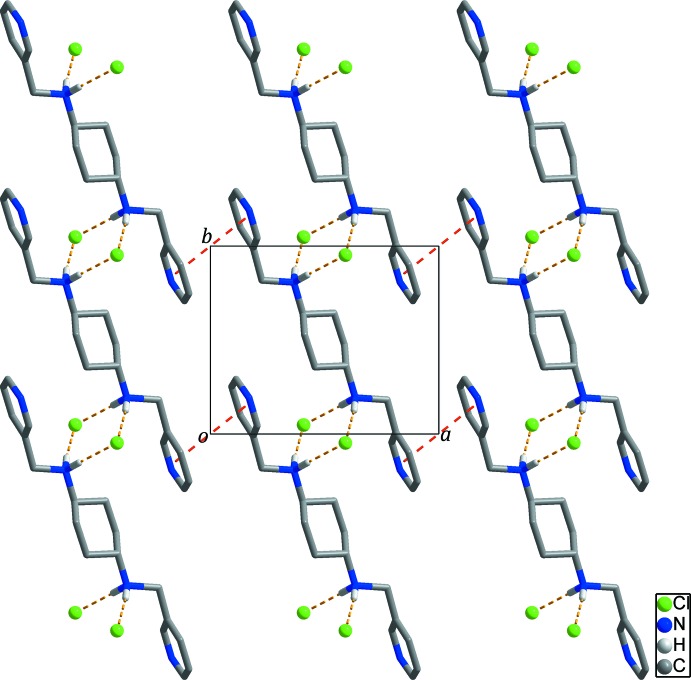
The two-dimensional sheet of the title salt formed through inter­molecular N^+^—H⋯Cl^−^ hydrogen bonds (yellow dashed lines) between the dications and the chloride anions and π–π stacking inter­actions (red dashed lines) between the pyridine rings of dications. H atoms not involved in inter­molecular inter­actions have been omitted for clarity.

**Figure 3:**
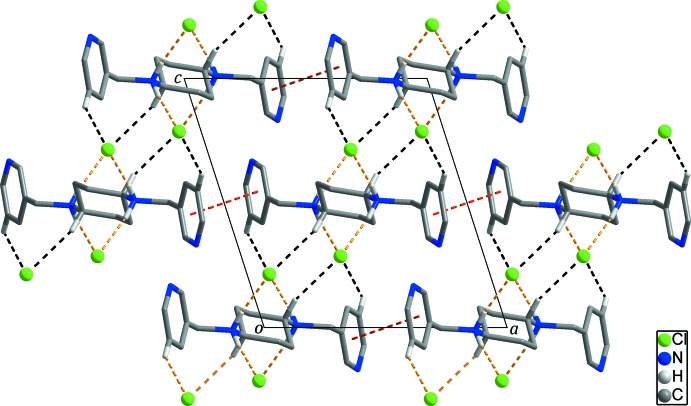
The three-dimensional supra­molecular network of the title salt formed through inter­molecular C—H⋯Cl^−^ hydrogen bonds (black dashed lines) between the two-dimensional sheets constructed by inter­molecular N^+^—H⋯Cl^−^ hydrogen bonds (yellow dashed lines) and π–π stacking inter­actions (red dashed lines). H atoms not involved in inter­molecular inter­actions have been omitted for clarity.

In the crystal, N^+^–H⋯Cl^−^ hydrogen bonds, Table 1 ▸ (yellow dashed lines in Figs. 2 ▸ and 3 ▸), between the dications and the chloride anions lead to the formation of chains along the *b* axis. Adjacent chains are additionally connected through inter­molecular π–π stacking inter­actions [centroid-to-centroid distance = 3.8197 (8) Å] between the pyridine rings (red dashed lines in Figs. 2 ▸ and 3 ▸), resulting in the formation of a sheet extending parallel to the *ab* plane. These sheets are linked by weak C–H⋯Cl^−^ hydrogen bonds, Table 1 ▸ (black dashed lines in Fig. 3 ▸), between the dications and the chloride anions, forming a three-dimensional supra­molecular network.

## Database survey   

A search of the Cambridge Structural Database (Version 5.37, Feb 2016 with two updates; Groom *et al.*, 2016 ▸) revealed only a Co^II^ complex with the dication of the title salt as a ligand, namely *catena*-[bis­(*μ* ^2^-*N*,*N′*-bis­(pyridin-3-ylmeth­yl)cyclo­hexane-1,4-diaminium)(nitrato-*O,O′*)cobalt(II) penta­nitrate methanol solvate] (Lee & Lee, 2010 ▸). Each Co^II^ ion in this complex is six-coordinated by two O atoms of one nitrate anion and four N atoms of four dipyridyl-type dication ligands to form a distorted octahedral geometry.

## Synthesis and crystallization   

*N*,*N*-bis­(pyridin-3-yl­methyl­ene)cyclo­hexane-1,4-di­amine, prepared according to a literature method (Huh & Lee, 2007 ▸), was dissolved in ethanol, and then the pH was adjusted to 4–5 with 2 *M* hydro­chloric acid. The resultant mixture was left to evaporate slowly over several days, resulting in the formation of X-ray quality single crystals of the title salt.

## Refinement   

Crystal data, data collection and structure refinement details are summarized in Table 2 ▸. The position of the pyridine nitro­gen atom was determined by the difference in the displacement parameters. All C-bound H atoms were positioned geometrically [with *d*(C—H) = 0.95 Å for C*sp* ^2^—H, 0.99 Å for methyl­ene, 1.00 Å for methine H atoms] and were refined as riding with *U*_iso_(H) = 1.2*U*_eq_(C). The N-bound H atoms involved in hydrogen bonds were located in difference Fourier maps and refined freely [N—H = 0.891 (15) and 0.876 (16) Å].

## Supplementary Material

Crystal structure: contains datablock(s) I, New_Global_Publ_Block. DOI: 10.1107/S2056989016017205/hg5479sup1.cif

Structure factors: contains datablock(s) I. DOI: 10.1107/S2056989016017205/hg5479Isup2.hkl

CCDC reference: 1511616

Additional supporting information: crystallographic information; 3D view; checkCIF report

## Figures and Tables

**Table 1:** Hydrogen-bond geometry (Å, °)

*D*—H⋯*A*	*D*—H	H⋯*A*	*D*⋯*A*	*D*—H⋯*A*
N1—H1*A*⋯Cl1	0.891 (15)	2.237 (15)	3.1215 (10)	171.7 (12)
N1—H1*B*⋯Cl1^i^	0.876 (16)	2.287 (16)	3.1588 (10)	173.8 (13)
C3—H3⋯Cl1^ii^	1.00	2.76	3.7106 (11)	158
C8—H8⋯Cl1^iii^	0.95	2.76	3.6020 (13)	148

Symmetry codes: (i) 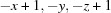; (ii) 
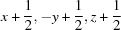
; (iii) 
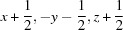
.

**Table 2:** Experimental details

Crystal data	
Chemical formula	C_18_H_26_N_4_^2+^·2Cl^−^
*M*_r_	369.33
Crystal system, space group	Monoclinic, *P*2_1_/*n*
Temperature (K)	173
*a*, *b*, *c* (Å)	10.4637 (2), 8.1942 (2), 11.2797 (2)
β (°)	107.812 (1)
*V* (Å^3^)	920.78 (3)
*Z*	2
Radiation type	Mo *K*α
μ (mm^−1^)	0.36
Crystal size (mm)	0.32 × 0.27 × 0.21

Data collection	
Diffractometer	Bruker APEXII CCD
Absorption correction	Multi-scan (*SADABS*; Bruker 2013 ▸)
*T*_min_, *T*_max_	0.671, 0.746
No. of measured, independent and observed [*I* > 2σ(*I*)] reflections	8881, 2303, 2118
*R*_int_	0.022
(sin θ/λ)_max_ (Å^−1^)	0.670

Refinement	
*R*[*F* ^2^ > 2σ(*F* ^2^)], *wR*(*F* ^2^), *S*	0.030, 0.083, 1.03
No. of reflections	2303
No. of parameters	117
H-atom treatment	H atoms treated by a mixture of independent and constrained refinement
Δρ_max_, Δρ_min_ (e Å^−3^)	0.29, −0.26

Computer programs: *APEX2* and *SAINT* (Bruker, 2013 ▸), *SHELXS97* and *SHELXTL* (Sheldrick, 2008 ▸), *SHELXL2014* (Sheldrick, 2015 ▸) and *DIAMOND* (Brandenburg, 2010 ▸).

## supplementary crystallographic information

### Crystal data

**Table:**

C_18_H_26_N_4_^2+^·2Cl^−^	*F*(000) = 392
*M**_r_* = 369.33	*D*_x_ = 1.332 Mg m^−^^3^
Monoclinic, *P*2_1_/*n*	Mo *K*α radiation, λ = 0.71073 Å
*a* = 10.4637 (2) Å	Cell parameters from 4678 reflections
*b* = 8.1942 (2) Å	θ = 2.3–28.4°
*c* = 11.2797 (2) Å	µ = 0.36 mm^−^^1^
β = 107.812 (1)°	*T* = 173 K
*V* = 920.78 (3) Å^3^	Block, colourless
*Z* = 2	0.32 × 0.27 × 0.21 mm

### Data collection

**Table:**

Bruker APEXII CCD diffractometer	2118 reflections with *I* > 2σ(*I*)
φ and ω scans	*R*_int_ = 0.022
Absorption correction: multi-scan (SADABS; Bruker 2013)	θ_max_ = 28.4°, θ_min_ = 2.3°
*T*_min_ = 0.671, *T*_max_ = 0.746	*h* = −12→14
8881 measured reflections	*k* = −9→10
2303 independent reflections	*l* = −15→15

### Refinement

**Table:**

Refinement on *F*^2^	0 restraints
Least-squares matrix: full	Hydrogen site location: mixed
*R*[*F*^2^ > 2σ(*F*^2^)] = 0.030	H atoms treated by a mixture of independent and constrained refinement
*wR*(*F*^2^) = 0.083	*w* = 1/[σ^2^(*F*_o_^2^) + (0.0443*P*)^2^ + 0.3189*P*] where *P* = (*F*_o_^2^ + 2*F*_c_^2^)/3
*S* = 1.03	(Δ/σ)_max_ < 0.001
2303 reflections	Δρ_max_ = 0.29 e Å^−^^3^
117 parameters	Δρ_min_ = −0.26 e Å^−^^3^

### Special details

**Table:**

Geometry. All esds (except the esd in the dihedral angle between two l.s. planes) are estimated using the full covariance matrix. The cell esds are taken into account individually in the estimation of esds in distances, angles and torsion angles; correlations between esds in cell parameters are only used when they are defined by crystal symmetry. An approximate (isotropic) treatment of cell esds is used for estimating esds involving l.s. planes.

### Fractional atomic coordinates and isotropic or equivalent isotropic displacement parameters (Å^2^)

**Table:**

	*x*	*y*	*z*	*U*_iso_*/*U*_eq_	
Cl1	0.40846 (3)	0.04783 (3)	0.28633 (2)	0.02513 (10)	
N1	0.63461 (9)	0.19285 (11)	0.51048 (9)	0.01829 (19)	
H1A	0.5756 (14)	0.1538 (18)	0.4416 (13)	0.021 (3)*	
H1B	0.6295 (14)	0.1263 (19)	0.5696 (14)	0.026 (4)*	
N2	0.83020 (13)	−0.19199 (14)	0.33231 (10)	0.0343 (3)	
C1	0.54173 (12)	0.64376 (14)	0.44414 (11)	0.0240 (2)	
H1C	0.6086	0.6937	0.5170	0.029*	
H1D	0.5352	0.7127	0.3704	0.029*	
C2	0.58760 (13)	0.47226 (14)	0.42261 (11)	0.0236 (2)	
H2A	0.5242	0.4254	0.3462	0.028*	
H2B	0.6773	0.4780	0.4106	0.028*	
C3	0.59394 (11)	0.36313 (13)	0.53315 (10)	0.0180 (2)	
H3	0.6618	0.4083	0.6089	0.022*	
C4	0.77439 (12)	0.18178 (15)	0.50226 (12)	0.0265 (3)	
H4A	0.8381	0.2237	0.5804	0.032*	
H4B	0.7819	0.2515	0.4330	0.032*	
C5	0.81211 (11)	0.00954 (14)	0.48115 (10)	0.0207 (2)	
C6	0.79449 (14)	−0.04487 (16)	0.36097 (12)	0.0298 (3)	
H6	0.7541	0.0278	0.2944	0.036*	
C7	0.88687 (13)	−0.29136 (15)	0.42730 (12)	0.0289 (3)	
H7	0.9154	−0.3959	0.4092	0.035*	
C8	0.90643 (13)	−0.25138 (16)	0.54997 (12)	0.0299 (3)	
H8	0.9454	−0.3275	0.6144	0.036*	
C9	0.86810 (13)	−0.09778 (16)	0.57749 (11)	0.0271 (3)	
H9	0.8801	−0.0667	0.6613	0.033*	

### Atomic displacement parameters (Å^2^)

**Table:**

	*U*^11^	*U*^22^	*U*^33^	*U*^12^	*U*^13^	*U*^23^
Cl1	0.02754 (17)	0.02485 (16)	0.02046 (15)	−0.00212 (10)	0.00355 (11)	0.00078 (9)
N1	0.0189 (5)	0.0152 (4)	0.0209 (4)	0.0013 (3)	0.0064 (4)	−0.0008 (3)
N2	0.0445 (7)	0.0312 (6)	0.0274 (5)	0.0077 (5)	0.0114 (5)	−0.0051 (4)
C1	0.0265 (6)	0.0173 (5)	0.0333 (6)	0.0025 (4)	0.0168 (5)	0.0029 (4)
C2	0.0291 (6)	0.0187 (5)	0.0291 (6)	0.0045 (4)	0.0177 (5)	0.0032 (4)
C3	0.0197 (5)	0.0141 (5)	0.0199 (5)	0.0019 (4)	0.0056 (4)	−0.0019 (4)
C4	0.0205 (6)	0.0209 (6)	0.0404 (7)	0.0008 (4)	0.0125 (5)	−0.0048 (5)
C5	0.0177 (5)	0.0197 (5)	0.0261 (5)	0.0011 (4)	0.0086 (4)	−0.0014 (4)
C6	0.0379 (7)	0.0274 (6)	0.0236 (6)	0.0089 (5)	0.0087 (5)	0.0031 (4)
C7	0.0281 (6)	0.0199 (6)	0.0407 (7)	0.0032 (5)	0.0133 (5)	−0.0032 (5)
C8	0.0289 (6)	0.0269 (6)	0.0340 (6)	0.0068 (5)	0.0097 (5)	0.0089 (5)
C9	0.0271 (6)	0.0319 (7)	0.0222 (5)	0.0042 (5)	0.0075 (4)	−0.0002 (5)

### Geometric parameters (Å, º)

**Table:**

N1—C4	1.4963 (14)	C3—C1^i^	1.5188 (15)
N1—C3	1.5033 (13)	C3—H3	1.0000
N1—H1A	0.891 (15)	C4—C5	1.5040 (16)
N1—H1B	0.876 (16)	C4—H4A	0.9900
N2—C6	1.3309 (17)	C4—H4B	0.9900
N2—C7	1.3319 (17)	C5—C9	1.3813 (17)
C1—C3^i^	1.5188 (15)	C5—C6	1.3848 (16)
C1—C2	1.5283 (15)	C6—H6	0.9500
C1—H1C	0.9900	C7—C8	1.3750 (18)
C1—H1D	0.9900	C7—H7	0.9500
C2—C3	1.5193 (15)	C8—C9	1.3847 (18)
C2—H2A	0.9900	C8—H8	0.9500
C2—H2B	0.9900	C9—H9	0.9500
			
C4—N1—C3	113.56 (9)	C1^i^—C3—H3	109.0
C4—N1—H1A	110.8 (9)	C2—C3—H3	109.0
C3—N1—H1A	109.0 (9)	N1—C4—C5	112.05 (9)
C4—N1—H1B	107.3 (10)	N1—C4—H4A	109.2
C3—N1—H1B	111.2 (10)	C5—C4—H4A	109.2
H1A—N1—H1B	104.6 (13)	N1—C4—H4B	109.2
C6—N2—C7	116.57 (11)	C5—C4—H4B	109.2
C3^i^—C1—C2	110.35 (9)	H4A—C4—H4B	107.9
C3^i^—C1—H1C	109.6	C9—C5—C6	117.55 (11)
C2—C1—H1C	109.6	C9—C5—C4	122.80 (11)
C3^i^—C1—H1D	109.6	C6—C5—C4	119.62 (11)
C2—C1—H1D	109.6	N2—C6—C5	124.49 (12)
H1C—C1—H1D	108.1	N2—C6—H6	117.8
C3—C2—C1	110.35 (9)	C5—C6—H6	117.8
C3—C2—H2A	109.6	N2—C7—C8	123.82 (12)
C1—C2—H2A	109.6	N2—C7—H7	118.1
C3—C2—H2B	109.6	C8—C7—H7	118.1
C1—C2—H2B	109.6	C7—C8—C9	118.55 (11)
H2A—C2—H2B	108.1	C7—C8—H8	120.7
N1—C3—C1^i^	108.79 (9)	C9—C8—H8	120.7
N1—C3—C2	110.50 (8)	C5—C9—C8	118.99 (11)
C1^i^—C3—C2	110.60 (9)	C5—C9—H9	120.5
N1—C3—H3	109.0	C8—C9—H9	120.5
			
C3^i^—C1—C2—C3	−57.46 (14)	C7—N2—C6—C5	−0.2 (2)
C4—N1—C3—C1^i^	−172.97 (9)	C9—C5—C6—N2	−1.4 (2)
C4—N1—C3—C2	65.43 (12)	C4—C5—C6—N2	176.74 (13)
C1—C2—C3—N1	178.12 (9)	C6—N2—C7—C8	1.7 (2)
C1—C2—C3—C1^i^	57.60 (14)	N2—C7—C8—C9	−1.5 (2)
C3—N1—C4—C5	179.29 (9)	C6—C5—C9—C8	1.50 (18)
N1—C4—C5—C9	−88.08 (14)	C4—C5—C9—C8	−176.57 (11)
N1—C4—C5—C6	93.89 (13)	C7—C8—C9—C5	−0.16 (19)

Symmetry code: (i) −*x*+1, −*y*+1, −*z*+1.

### Hydrogen-bond geometry (Å, º)

**Table:**

*D*—H···*A*	*D*—H	H···*A*	*D*···*A*	*D*—H···*A*
N1—H1*A*···Cl1	0.891 (15)	2.237 (15)	3.1215 (10)	171.7 (12)
N1—H1*B*···Cl1^ii^	0.876 (16)	2.287 (16)	3.1588 (10)	173.8 (13)
C3—H3···Cl1^iii^	1.00	2.76	3.7106 (11)	158
C8—H8···Cl1^iv^	0.95	2.76	3.6020 (13)	148

Symmetry codes: (ii) −*x*+1, −*y*, −*z*+1; (iii) *x*+1/2, −*y*+1/2, *z*+1/2; (iv) *x*+1/2, −*y*−1/2, *z*+1/2.
